# An integrated multi-omic approach demonstrates distinct molecular signatures between human obesity with and without metabolic complications: a case–control study

**DOI:** 10.1186/s12967-023-04074-x

**Published:** 2023-03-29

**Authors:** Fayaz Ahmad Mir, Raghvendra Mall, Ehsan Ullah, Ahmad Iskandarani, Farhan Cyprian, Tareq A. Samra, Meis Alkasem, Ibrahem Abdalhakam, Faisal Farooq, Shahrad Taheri, Abdul-Badi Abou-Samra

**Affiliations:** 1 Qatar Metabolic Institute, Academic Health System, Hamad Medical Corporation, PO Box 3050, Doha, Qatar; 2 Qatar Computational Research Institute (QCRI), Hamad Bin Khalifa University, Doha, Qatar; 3 Department of Immunology, St. Jude Children’s Research Hospital, Memphis, USA; 4 College of Medicine, QU Health, Qatar University, Doha, Qatar; 5 National Obesity Treatment Center, Hamad Medical Corporation, Doha, Qatar; 6 Weil Cornell Medicine – Qatar, Doha, Qatar; 7 Biotechnology Research Center, Technology Innovation Institute, P.O. Box 9639, Abu Dhabi, United Arab Emirates

## Abstract

**Objectives:**

To examine the hypothesis that obesity complicated by the metabolic syndrome, compared to uncomplicated obesity, has distinct molecular signatures and metabolic pathways.

**Methods:**

We analyzed a cohort of 39 participants with obesity that included 21 with metabolic syndrome, age-matched to 18 without metabolic complications. We measured in whole blood samples 754 human microRNAs (miRNAs), 704 metabolites using unbiased mass spectrometry metabolomics, and 25,682 transcripts, which include both protein coding genes (PCGs) as well as non-coding transcripts. We then identified differentially expressed miRNAs, PCGs, and metabolites and integrated them using databases such as mirDIP (mapping between miRNA-PCG network), Human Metabolome Database (mapping between metabolite-PCG network) and tools like MetaboAnalyst (mapping between metabolite-metabolic pathway network) to determine dysregulated metabolic pathways in obesity with metabolic complications.

**Results:**

We identified 8 significantly enriched metabolic pathways comprising 8 metabolites, 25 protein coding genes and 9 microRNAs which are each differentially expressed between the subjects with obesity and those with obesity and metabolic syndrome. By performing unsupervised hierarchical clustering on the enrichment matrix of the 8 metabolic pathways, we could approximately segregate the uncomplicated obesity strata from that of obesity with metabolic syndrome.

**Conclusions:**

The data suggest that at least 8 metabolic pathways, along with their various dysregulated elements, identified via our integrative bioinformatics pipeline, can potentially differentiate those with obesity from those with obesity and metabolic complications.

**Supplementary Information:**

The online version contains supplementary material available at 10.1186/s12967-023-04074-x.

## Introduction

Obesity is a chronic progressive relapsing disease occurring when excessive fat accumulation exerts a deleterious effect on health [1]. Obesity can have multiple adverse health consequences including the development of the metabolic syndrome, characterized by insulin resistance, dyslipidemia, hypertension, and/or type 2 diabetes mellitus. The metabolic syndrome, in turn, increases the risk of atherosclerotic cardiovascular disease such as coronary artery disease, stroke and peripheral artery disease [2]. There is growing interest in defining obesity phenotypes to understand the relationship between excess adiposity and metabolic health. A subgroup, termed “Metabolically Unhealthy Obesity” phenotype (obesity with metabolic syndrome or OBM) develop metabolic complications while another subgroup, termed as “Metabolically Healthy Obesity” (obesity only or OBO), do not demonstrate or progress to the metabolic syndrome, even in later life [3–9].

The various definitions of obesity phenotypes and various disease outcomes studies have resulted in diverse findings. In particular, there is controversy regarding cardiovascular outcomes in OBO individuals; either a similar risk profile or a greater risk profile than healthy weight individuals has been reported [5, 10–16]. A lower risk profile for OBO compared to OBM has been observed. There have been suggestions that OBO status is transient, progressing to OBM; nevertheless, perhaps, the OBO phenotype is associated with longer protection from metabolic deterioration. One potential contributing factor to the development of metabolic consequences in OBM is body fat distribution [17]. Central abdominal adiposity and accompanied high liver fat content have been linked to the OBM phenotype, whereas subcutaneous adiposity has been associated with the OBO phenotype [18, 19]. Apart from fat distribution, the potential pathways mediating adiposity and metabolic phenotypes include alterations in adipogenesis, adipose tissue function and inflammation, angiogenesis, immune dysregulation, and mitochondrial dysfunction [20].

The study of OBO and OBM phenotypes provides insight into the biological pathways differentially affected by excess body fat in each phenotype allowing prognostication and development of future treatments to target specific pathways to protect against metabolic dysfunction. In this work, we used an integrative bioinformatics pipeline to identify enriched metabolic pathways by integrating data from transcriptional signatures, microRNAs and circulating metabolites, to obtain a more holistic picture of the differential enrichment of metabolic pathways between OBO and OBM.

## Materials and methods

### Study design

The study was a cross-sectional study approved by the institutional review board (IRB) of Hamad Medical Corporation (HMC, IRB protocol #16245/16) and all participants provided written informed consent. Participants were recruited at the Qatar Metabolic Institute, Hamad Medical Corporation, Doha, Qatar. After signing the informed consent, they were invited to the study site for anthropometric measurements and collection of fasting blood samples between 7 and 9 A.M. after 12–14 h of fasting.

Participants included both men and women aged 18 to 65 years with body mass index (BMI) ≥ 35 kg/m^2^ with or without the metabolic syndrome but without other chronic disease or terminal illness. Participants were classified into two groups, based on presence of the components of the metabolic syndrome (obesity only [OBO] versus obesity with metabolic syndrome [OBM]) using the International Diabetes Federation (IDF) metabolic syndrome criteria: BMI > 30 kg/m^2^ or waist circumference above the ethnic threshold (102 cm for men and 88 cm for women) PLUS any 2 of: triglycerides ≥ 150 mg/dL (1.7 mmol/L), HDL-cholesterol < 40 mg/dL (1.03 mmol/L) in men or < 50 mg/dL (1.29 mmol/L) in women, blood pressure ≥ 130 / 85 mmHg, and fasting blood glucose ≥ 110 mg/dL (5.6 mmol/L).

### Blood chemistry assays

For serum collection, whole blood was collected via BD Vacutainer Serum Separation Tubes (BD Biosciences, Franklin Lakes, NJ, USA). Blood samples were kept at room temperature for 30–60 min, and then centrifuged at 3000*g* for 10 min. Following centrifugation, serum was separated, aliquoted and immediately stored at − 80 °C for further use. Blood biochemistry measurements were performed at the HMC clinical laboratory. Measurements included HbA1c via Turbidimetric Inhibition Immunoassay (TINIA Roche Diagnostics, Mannheim, Germany), glucose by enzymatic reference method with hexokinase (Cobas 6000, Roche Diagnostics International, Switzerland), and triglycerides, total and HDL cholesterol by calorimetric assays.

### RNA isolation and quality control

Whole blood (2.5 mL) was collected into PaXgene Blood RNA Tubes (PreAnalytix). The tubes were inverted 8–10 times then placed at room temperature for 2–3 h and then frozen at − 80 °C for storage. The samples were thawed overnight, then total RNA was isolated with a PAXgene Blood RNA Kit including the DNase Set (Qiagen). The concentrations and purity of the RNA samples were evaluated spectrophotometrically (Nanodrop ND-1000, Thermo, Wilmington, DE USA). The RNA isolation process was validated by analyzing the integrity of several RNAs with the RNA 6000 Nano Chip Kit (Agilent). The presence of the small RNA fraction was confirmed by the Agilent Small RNA Kit (Agilent).

### MicroRNA (miRNA) profiling

The expression levels of 754 miRNAs were determined using the TaqMan OpenArray Human MicroRNA panels (PN: 4,470,189; Life Technologies Foster City, CA, USA) on a QuantStudio 12 K Flex instrument. For all experimental groups, 3 µL (10 ng) of total RNA was used for reverse transcription (RT) reactions using MegaPlex RT Primers Human Pool Set v3.0 (PN: 4,444,745; Pool A v2.1 and Pool B v3.0) according to the manufacturer’s optimized protocol for low sample input for profiling human microRNA, using the OpenArray platform on BioRad c1000 Touch thermal cycler. No-template controls were included. Pre-amplification of RT products was performed using 5 µL RT reaction combined with the matching Megaplex PreAmp Primer Pool A v2.1 or B v3.0 and amplified using the thermal cycler (Applied biosystems). The pre-amplified products were diluted 1:40 in 0.1 × TE pH 8. For each experimental set, 10 µL of the diluted products were combined to give a total of 40 µL pooled sample. For both Pool A and Pool B groups, 22.5 µL of the pooled products were combined with an equivalent volume of TaqMan OpenArray Real-Time Master Mix and aliquoted into a 96 well plate. Then, 5 µL from each well were then transferred into a 384 well plate for loading onto OpenArray plates using an AccuFill robotic system. The OpenArray plates were run on a QuantStudio 12 K Flex instrument (Life Technologies) and the raw data files were imported and analyzed using the DataAssist software (Life Technologies). Failed reactions were excluded from analysis and undetermined CT values for sample sets determined to have good amplifications were assigned a threshold value of 40, defining low abundance or absence of miRNA expression. Global mean normalization was used to calculate relative fold change for the miRNA expression.

### Transcriptomic profiling

A Clariom D Assay-Human system (Applied Biosystems) containing 47,231 expressions and 770 control probes was used for transcriptomics. In brief, 100 ng of total RNA was reverse-transcribed into cDNA using GeneChip™ WT PLUS Reagent Kit (Applied biosystems); 5.5 µg of ss-cDNA was fragmented and labeled then hybridized for 16 h to the GeneChip™ cartridge array in a GeneChip Hybridization Oven 640. Arrays were washed and stained in a GeneChip Fluidics Station 450 and scanned in a GeneChip Scanner 3000 7G. The signal values were evaluated using the Affymetrix® GeneChip™ Command Console software.

The CEL files containing the transcriptional signatures were further analyzed using the affy v1.74.0 [21], oligo v1.60.0 [22], clariomdhumantranscriptcluster.db v8.8.0 [23], pd.clariom.d.human v3.14.1 [24], affycoretools 1.68.1 [25] packages in R. The raw transcriptional data was read through the ‘read.celfiles’ function in the oligo package. The quality control steps were performed using the ‘rma’ function in the oligo package; these include background correction, quantile normalization, and a log transformation of the transcript counts. The background transcript was filtered out using the ‘getMainProbes’ function in the affy coretools package. The ‘annotateEset’ function in the affy package was used by passing the transcriptional matrix along with the clariomhumantranscriptcluster.db for mapping the probe IDs to their corresponding ENTREZ IDs and Gene Symbols. By selecting only those probe IDs which can map to a corresponding ENTREZ ID, we obtained the final transcriptional matrix comprising 25,682 genes (including PCGs as well as non-coding genes) with their corresponding expression values for the 39 participant profiles.

### Metabolomic profiling

Metabolomic profiling was performed using established protocols at Metabolon, Durham, NC, USA. Sample accession, sample preparation, sample processing and QC generation was done at the Anti-Doping Laboratory—Qatar (ADLQ) with final review of QC data and curation by Metabolon. Samples were prepared using the automated MicroLab STAR® system from Hamilton Company. Several recovery standards were added prior to the first step in the extraction process for QC purposes. Proteins were precipitated with methanol under vigorous shaking for 2 min (Glen Mills GenoGrinder 2000) followed by centrifugation. The resulting extract was divided into five fractions, the sample extracts were stored overnight under nitrogen before preparation for analysis. The study utilized Waters ACQUITY UPLC, and a Thermo Scientific Q-Exactive high resolution/accurate mass spectrometer interfaced with a heated electrospray ionization (HESI-II) source and Orbitrap mass analyzer operated at 35,000 mass resolution. The extracted five fractions were reconstituted in solvents compatible to each of the four methods. Two fractions were analyzed by two separate reverse phases (RP)/UPLC-MS/MS methods with positive ion mode electrospray ionization (ESI), one was analyzed by RP/UPLC-MS/MS with negative ion mode ESI, one analyzed by hydrophilic interaction chromatography (HILIC)/UPLC-MS/MS with negative ion mode ESI, and one sample was reserved for backup. The analysis was done on two types of columns, C18 column (Waters UPLC BEH C18-2.1 × 100 mm, 1.7 µm) and HILIC column (Waters UPLC BEH Amide 2.1 × 150 mm, 1.7 µm). Raw data were extracted, peak-identified and quality control processed using Metabolon’s hardware and software. Compounds were identified by comparison to library entries of purified standards or recurrent unknown entities. A total of 1069 metabolites were profiled; 174 unnamed metabolites were removed leaving 895 metabolites for the rest of the analysis.

Several quality control steps were used to ensure data quality. Assuming values to be missing at random, 191 metabolites having less than 80% non-missing values (more than 20% missing values per metabolite due to biological and/or technical reasons) were removed [26] resulting in 704 metabolites for downstream analysis. Missing values in the remaining data were imputed by replacing the missing values for each metabolite with the minimum value detected for the metabolite. Outliers in samples were identified using principal component analysis (PCA) [27, 28] and only one sample was removed.

### Data analysis

Statistical characteristics of clinical measurements were calculated by comparing the OBO and OBM samples using R [29]. Student’s t-test was used to calculate p-value of the normally distributed measurement; group variance in this case was tested using F-test. For remaining measurements, the Mann–Whitney test was used. The normality of the measurements was tested using the Anderson–Darling test. P-values were not corrected for false discovery rate (FDR) due to small sample size.

### MiRNA – PCG interaction network

We utilized the microRNA Data Integration Portal, mirDIP v4.1 (http://ophid.utoronto.ca/mirDIP/), which consists of over 152 million human microRNA–target predictions collected from 30 different resources [30–32]. The mirDIP integrative score was constructed by taking a statistical consensus from the predictions available through myriad resources and was assigned to each unique miRNA-target interaction to provide a unified measure of confidence. The integrated scores range, 0 to 1, was used; higher scores correspond to stronger evidence of potential interaction between miRNA and target protein coding gene (PCG); the target PCGs were thus identified.

By focusing on interactions between differentially expressed miRNAs (OBM vs OBO) and PCGs, we initially have a reduced set of 53,625 miRNA-target gene interactions (MTIs). Further, focusing on those MTIs, where the number of available sources for interaction was >  = 9 (including reporter assay, western blot, microarray, NGS etc.) and the integrated score was >  = 0.25, resulted in 17,797 unique MTIs. Additionally, for our use case, we removed those MTIs where the target PCG was not differentially expressed. This resulted in a miRNA-PCG network consisting of 457 nodes (55 DE-miRNAs and 402 differentially expressed protein coding genes (DE-PCGs)) and 991 miRNA-PCG interaction edges.

### Metabolite set enrichment analysis

Metabolite set enrichment analysis (MSEA) or pathway enrichment analysis was performed using the online version of Metaboanlyst 5.0 (https://www.metaboanalyst.ca/). Data file (comma separated values) was prepared using R for Metaboanlyst containing samples, disease status, compounds IDs, and values of metabolites for each sample. The compounds were matched using HMDB IDs provided by Metabolon. For pathway enrichment analysis, autoscaling was used to normalize each variable to be mean centered with a unit variance. KEGG human metabolic pathways corresponding to 84 metabolite sets were used as the reference pathways for the enrichment analysis. Metabolite sets having less than two metabolites in our dataset were filtered out.

### Single sample gene set enrichment

To obtain an estimate of enrichment of the metabolic pathways, identified through our integrative multi-omics analysis framework, for each sample, we used the Gene Set Variation Analysis (GSVA) function in R [33–35]. GSVA is a non-parametric, unsupervised technique, used to estimate gene set enrichment scores as a function of genes inside and outside the gene set analogously to a competitive gene set test. In our scenario, the gene set comprises the list of DE-PCGs which are participating as kinases/enzymes in the metabolic pathways. Mathematically, this can be represented as G(p_i_) = (g_1_, …, g_k_), where G(p_i_) represents the gene set corresponding to i^th^ metabolic pathway and consists of k DE-PCGs with respect to the phenotype of interest. Each of these G(p_i_) is passed to GSVA along with the transcriptomic expression matrix keeping all other parameters to default settings to obtain the single sample enrichment scores for each G(p_i_). Once the enrichment score for each G(p_i_) is obtained, an unsupervised hierarchical clustering was then performed to determine whether the enrichment scores of the metabolic pathways can distinguish OBO from OBM using the ‘hclust’ function with ‘ward.D’ method in R. This helps to narrow down the targetable metabolic pathways which are differentially enriched with respect to the phenotype.

### Integrative bioinformatics pipeline

**Fig. 1:**
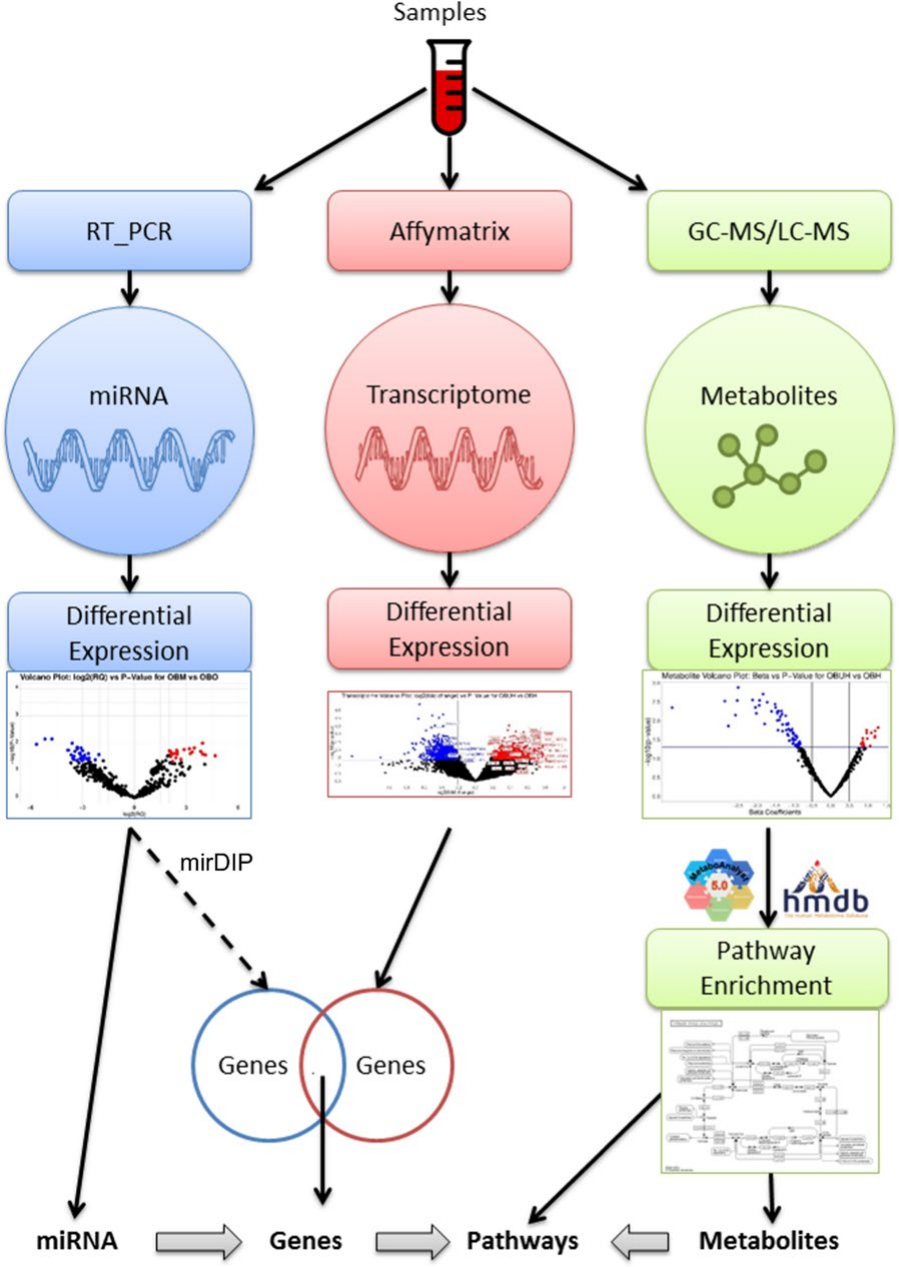
Comprehensive information about multiple dysregulated elements involved in the metabolic pathways that are differentially enriched between OBO vs OBM

The myriad omics profiles including miRNA through RT-PCR, transcriptional signatures through Affymetrix and metabolic profiles through LC–MS technique are illustrated in Fig. 1. The differences between OBO and OBM subjects for each omics type are deduced using a Bayesian statistical framework, namely LIMMA [36] (limma package v3.52.4 in R), which identifies differentially expressed miRNAs, PCGs and metabolites at a p-value threshold of 0.05 (no FDR correction owing to small sample size).

We then perform metabolite set enrichment analysis resulting in enriched metabolic pathways. We used the mirDIP database to obtain information about MTIs. For our use case, we focused on those target PCGs which are involved in the enriched metabolic pathways and were also differentially expressed with respect to the phenotype (p-value ≤ 0.05, LIMMA). Thus, we concentrated on the DE-PCGs which act as enzymes/kinases in these enriched metabolic pathways. For each of these DE-PCGs, we obtained the corresponding miRNAs (also differentially expressed) that were known to regulate the expression of these genes with high integrated scores using the mirDIP database. A snapshot of the entire workflow is depicted in Fig. 1 and Additional file 1: Figure S1.

## Results

### Clinical characteristics

The clinical characteristics of the sample are summarized in Table 1. As OBO and OBM participants were matched for age and BMI; the mean age and BMI for OBO and OBM groups were not significantly different. The mean ages were 38.1 and 40.5 years and mean BMIs 40.0 and 39.6 kg/m^2^, for OBO and OBM, respectively. There were 11 women and 7 men in the OBO group and 9 women and 12 men in the OBM group. The clinical traits that were significantly different between the two groups include HBA1c (p = 0.002), fasting triglycerides (p = 0.001), high-density lipoprotein cholesterol (HDL-cholesterol, p = 0.008), fasting glucose (p = 0.009) and insulin levels (p = 0.05). Other important clinical traits which were not significantly different between the two sets include clinical variables such creatinine, total cholesterol, and low-density lipoprotein cholesterol (LDL-cholesterol) levels. A greater percentage of the OBM group reported cigarette smoking.

**Table 1:** Clinical and biochemical traits of the study subjects. OBO, obesity only and OBM, obesity with metabolic syndrome. Significance was determined by the Student’s t-test

Feature	OBO N = 18	OBM N = 21	P Value
Age (years)	38.1 ± 4.2	40.5 ± 7.3	0.283
Females (N; %)	11 (61%)	9 (42.9%)	0.415
Height (cm)	167.4 ± 11.9	170.8 ± 9.6	0.370
Weight (kg)	113.4 ± 19.6	110.9 ± 27.6	0.782
BMI (kg/m^2^)	40.0 ± 4.5	39.6 ± 3.0	0.746
Smoking (%)	6.0	33.0	0.027
HBA1c (%)	5.5 ± 0.27	7.02 ± 1.9	0.002
Fasting triglycerides (mmol/L)	1.39 ± 0.48	2.65 ± 1.52	0.001
Total Cholesterol (mmol/L)	4.9 ± 1.1	4.8 ± 1.1	0.855
LDL-Cholesterol (mmol/L)	2.8 ± 1.3	2.6 ± 1.1	0.728
HDL-Cholesterol (mmol/L)	1.5 ± 0.7	1.0 ± 0.3	0.008
Fasting glucose (mmol/L)	5.2 ± 0.6	7.4 ± 3.4	0.009
Creatinine (μmol/L)	67.5 ± 14.1	65.3 ± 14.1	0.563
Fasting insulin (miU/mL)	19.0 ± 13.3	27.6 ± 13.2	0.053
C-reactive protein (mg/L)	12.8 ± 12.5	7.1 ± 4.5	0.064
Alanine transaminase (U/L)	20.7 ± 11.6	36.5 ± 35.1	0.063
Aspartate aminotransferase (U/L)	18.8 ± 9.6	23.6 ± 15.0	0.251

### Enrichment analysis of metabolic pathways

**Fig. 2:**
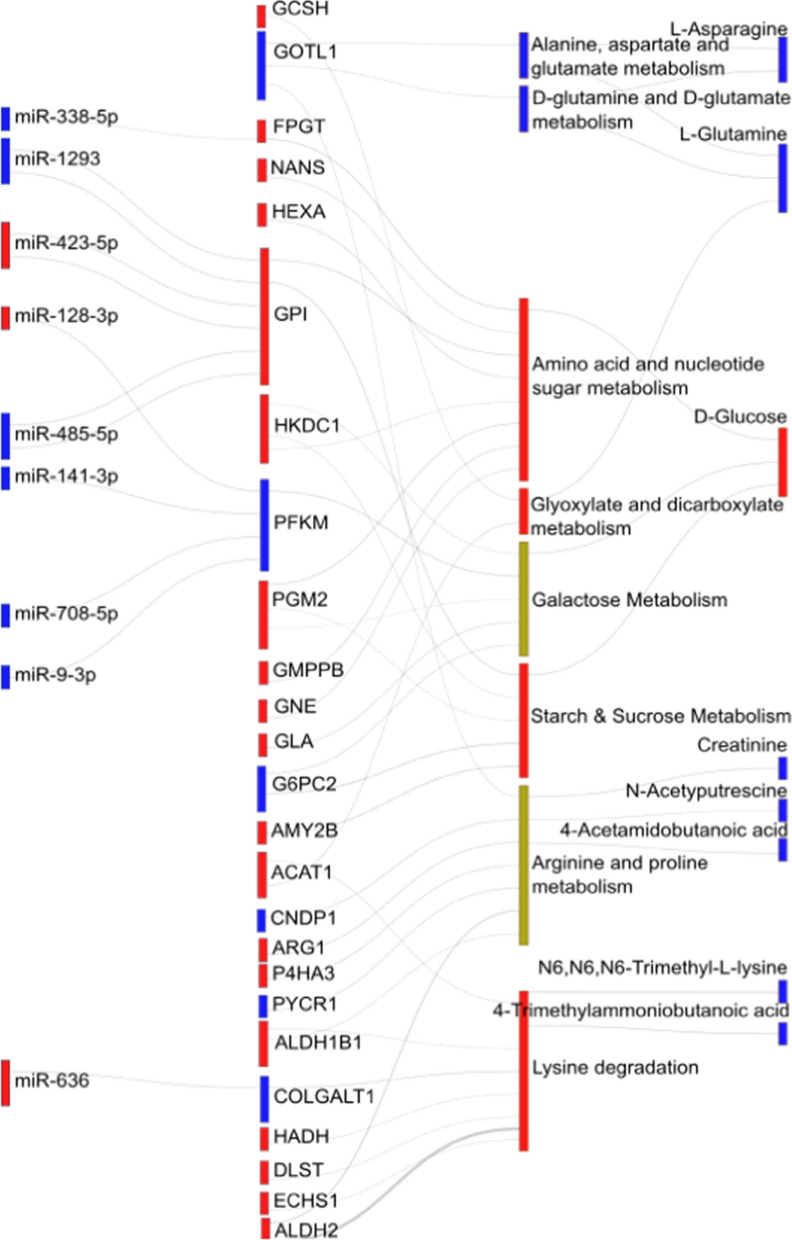
Sankey Plot highlighting interactions between differentially expressed regulatory elements and their associated metabolic enriched pathways. The ‘red’ color represents over-expression of a regulatory element while the ‘blue’ color corresponds to down-regulation of the regulatory element in OBM samples in comparison to the OBO samples, respectively

The results of metabolic pathway enrichment analysis using metabolite signatures in the cohort are shown in Table 2 and Fig. 2 and. Out of 61 metabolic pathways, 12 were significantly (p ≤ 0.1) enriched. Due to the small sample number, the p-values were relatively high and statistic Q was high; however, no FDR correction was used for p-values. The enriched pathways included Lysine degradation (Q = 11.62, p = 0.003); Amino sugar and nucleotide sugar metabolism (Q = 14.15, p = 0.005); Arginine and proline metabolism (Q = 6.15, p = 0.015); Fructose and mannose metabolism (Q = 10.85, p = 0.017); Galactose metabolism (Q = 8.53, p = 0.02); Starch and sucrose metabolism (Q = 8.35, p = 0.05); D-Glutamine and D-glutamate metabolism (Q = 7.45, p = 0.05); Alanine, aspartate and glutamate metabolism (Q = 4.80, p = 0.078); Fatty acid elongation (Q = 8.63, p = 0.078); Steroid biosynthesis (Q = 7.91, p = 0.09); Nicotinate and nicotinamide metabolism (Q = 5.44, p = 0.09); and Glyoxylate and dicarboxylate metabolism (Q = 5.09, p = 0.09).

**Table 2:**
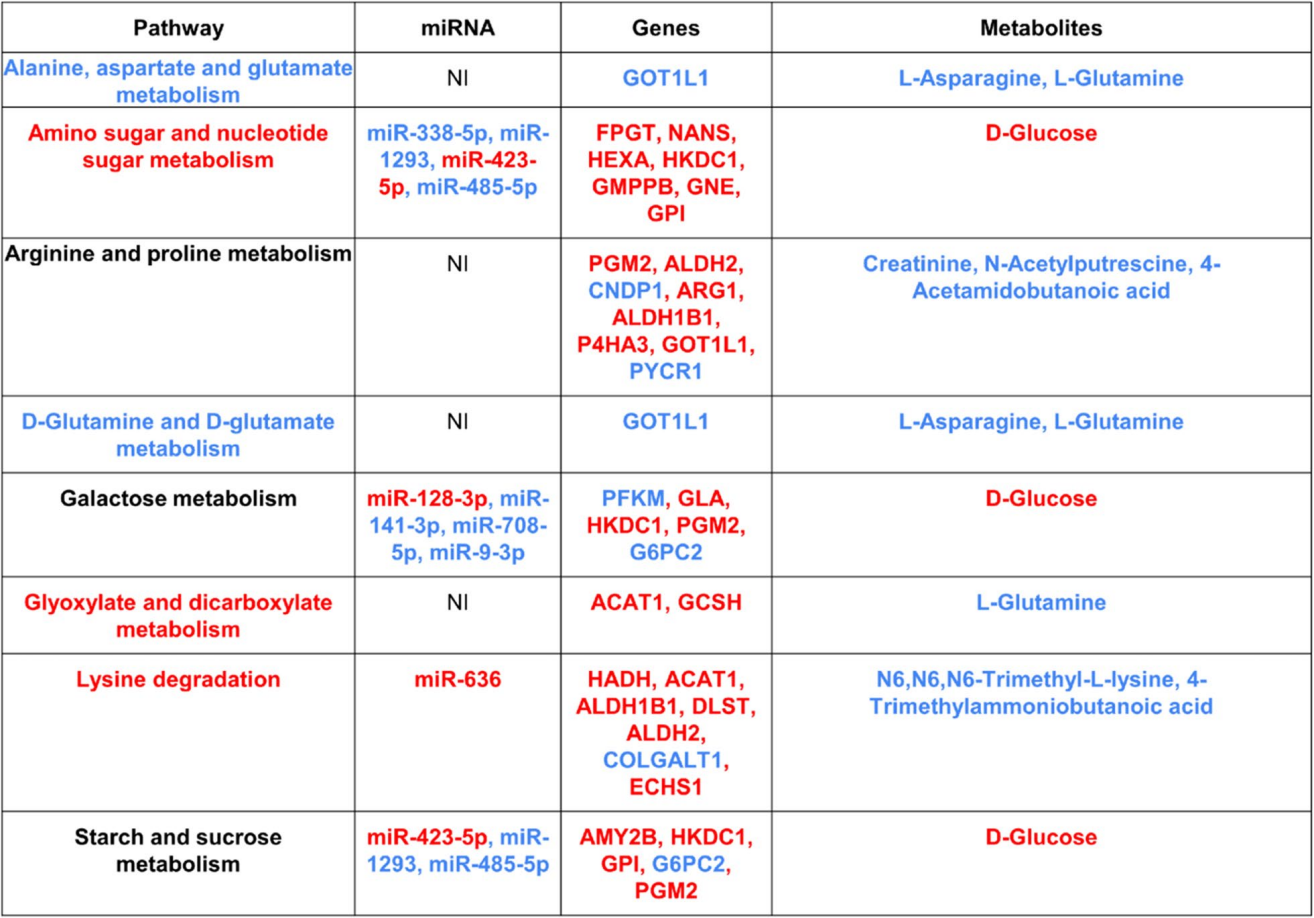
List of differentially expressed miRNA, genes and metabolites associated with enriched pathways. Here ‘NI’ refers to ‘non-identified’ or no miRNAs identified. The ‘red’ colored regulatory elements are significantly up-regulated, and the ‘blue’ colored regulatory elements are significantly down-regulated based on our framework

### Regulatory elements in enriched metabolic pathways

The metabolic pathway enrichment analysis identified 12 enriched pathways. Out of these 12 metabolic pathways, there were only 8 pathways which were associated with differentially expressed metabolites and include differentially expressed PCGs as kinases in these pathways. Hence, we focused on these 8 metabolic pathways for further analysis. The myriad regulatory elements involved in those pathways including the miRNAs, PCGs and metabolites were illustrated in Fig. 2 as a Sankey plot and in Table 2. A total of 8 differentially expressed (DE) metabolites along with 25 DE-PCGs (p ≤ 0.05) participating as kinases in at least one of these metabolic pathways, are illustrated; together with 9 upstream DE miRNAs (p ≤ 0.05) showing 12 out of 991 interaction edges between the DE-miRNA – DE-PCGs and were highlighted in Fig. 2.

The 8 enriched metabolic pathways include amino sugar and nucleotide sugar metabolism, alanine, aspartate and glutamate metabolism, arginine and proline metabolism, D-glutamine and D-glutamate metabolism, galactose metabolism, glyoxylate and dicarboxylate metabolism, lysine degradation and starch and sucrose metabolism (Table 2). A detailed list of DE-MiRNAs, DE-PCGs, metabolites along with their direction of regulation in the enriched pathways were also highlighted (Table 2). For the majority of the metabolic pathways, the DE-miRNAs were down-regulated in OBM samples (‘blue’ in Fig. 2) while their corresponding target DE-PCGs were primarily up-regulated [37] except PFKM, which was significantly down-regulated. Interestingly, the metabolic pathways D-glutamine and D-glutamate metabolism and alanine, aspartate and glutamate metabolism along with its associated DE-PCG, GOT1L1, and metabolites, L-Asparagine and L-Glutamine were all significantly down-regulated in OBM samples as compared to OBO.

### GSVA pathway enrichment and clinical implications

**Fig. 3:**
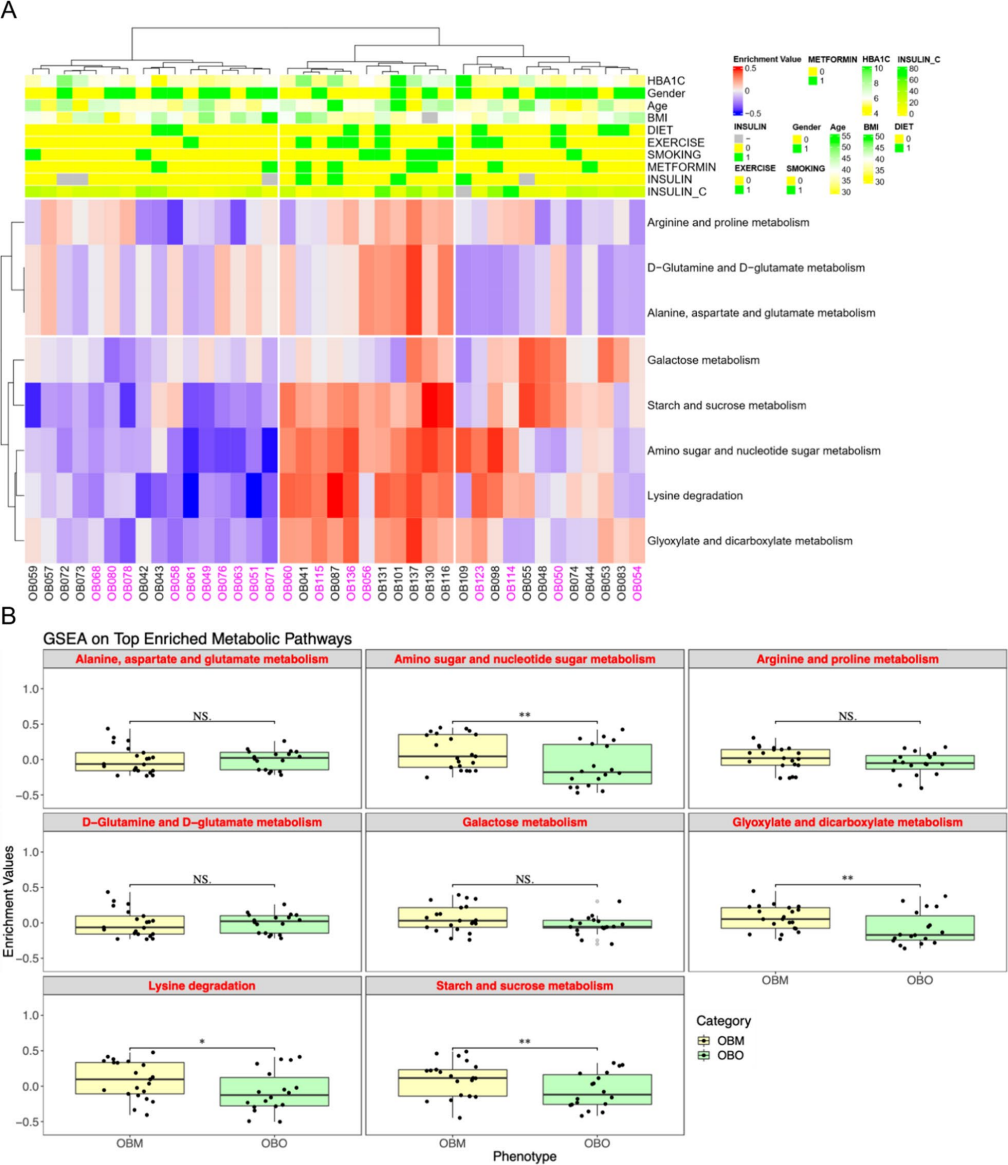
Clinical implications of enriched metabolic pathways and their correlation with clinical traits. Here the ‘purple’ colored column labels correspond to OBO samples while the ‘black’ colored column labels represent the OBM samples in **A**. p-values in **B** were obtained through a t-test procedure and ‘*’ correspond to p-value ≤ 0.05 and p-value > 0.01, ‘**’ correspond to p-value < 0.01 and p-value > 0.001 and ‘NS’ stand for ‘not-significant’ p-value

We performed single sample gene set enrichment analysis (GSVA) using the gene expression of the DE-PCGs involved in the 8 metabolic pathways to obtain their enrichment values as shown in Fig. 3 (rows corresponding to metabolic pathways and columns represent the participant samples). Using an unsupervised clustering technique, the participant data was segregated into 2 clusters (C1 and C2) corresponding to the OBO and OBM strata respectively. The majority of the 8 metabolic pathways were significantly positively enriched in the cluster C2 while they were negatively enriched in the cluster C1. Thus, the enrichment of these 8 metabolic pathways could be used in a predictive framework to potentially distinguish OBM from OBO.

Moreover, as observed in Fig. 3A, we highlighted several of the clinical traits including age, sex, BMI, diet and exercise status, blood glucose levels and medication information. Interestingly, within the OBM C2 cluster, two predominant sub-clusters could be distinguished based on blood glucose and insulin levels; in one OBM sub-cluster there were more subjects with higher blood glucose and insulin levels (potentially diabetes); and at the same time, they reported greater exercise and smoking with higher enrichment values for the 8 metabolic pathways.

Figure 3B provides a comprehensive comparison of the enrichment values between the OBO and OBM group for each of the 8 metabolic pathways of interest and shows pathways that were most significantly differentially enriched between the OBO and OBM such as amino sugar and nucleotide sugar metabolism, glyoxylate and dicarboxylate metabolism, lysine degradation and starch and sucrose metabolism.

**Fig. 4:**
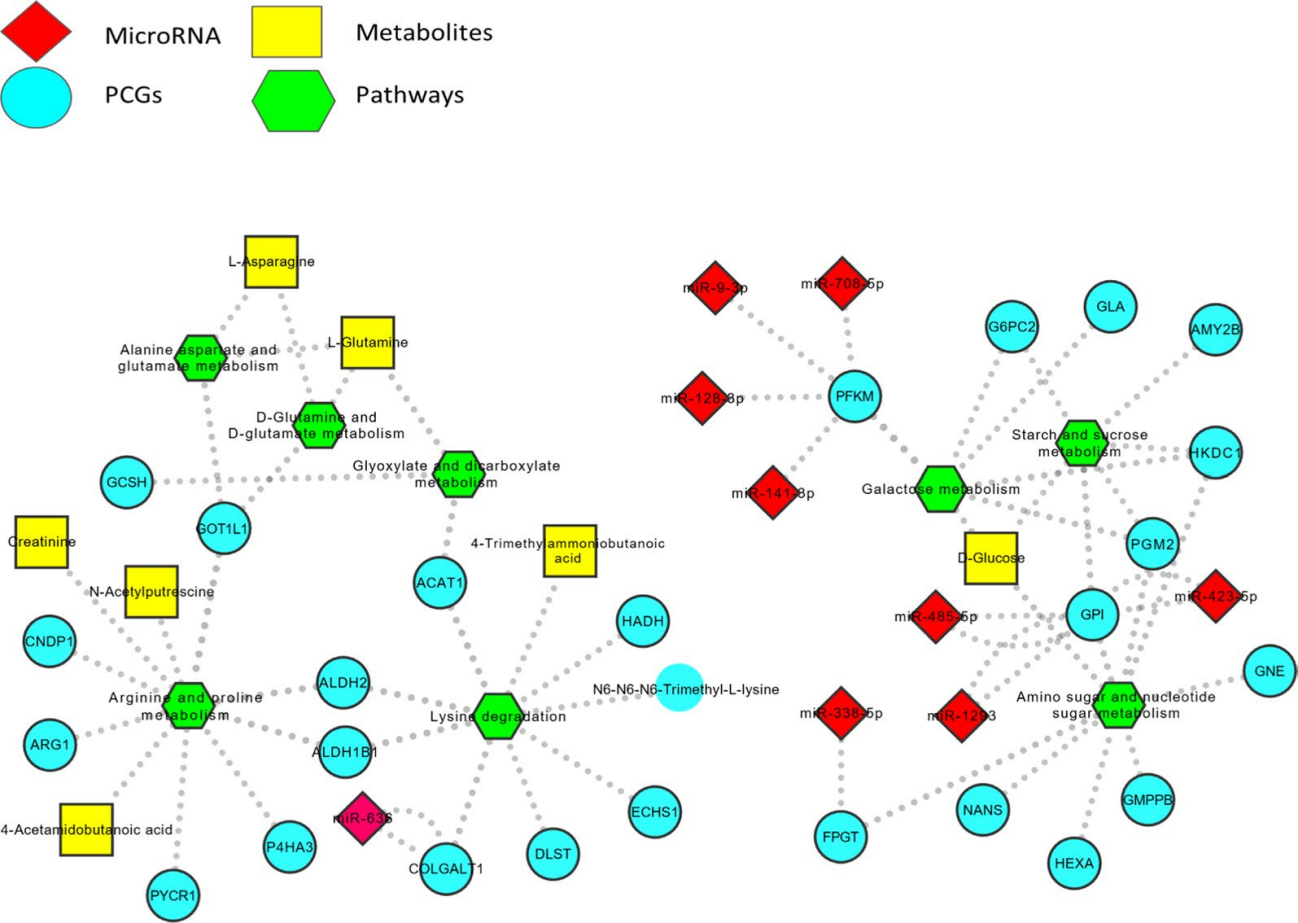
Network representation of the interaction between different regulatory elements associated with the enriched metabolic pathways

A network-based representation of the myriad regulatory elements involved in the 8 enriched metabolic pathways was illustrated in Fig. 4. We observed two clear clusters of regulatory elements with one cluster comprising alanine, aspartate and glutamate metabolism, D-glutamine and D-glutamate metabolism, glyoxylate and dicarboxylate metabolism, lysine degradation and arginine and proline metabolism pathways along with primarily DE-PCGs and differentially expressed metabolites. There was only one DE-miRNA (miR-636) associated in this cluster. The other cluster comprising galactose metabolism, starch and sugar metabolism and amino sugar and nucleotide sugar metabolism included the remaining 9 DE-miRNAs and DE-PCGs with the inclusion of D-Glucose as the lone differentially expressed metabolite. All metabolic pathways in the second cluster were associated with sugar metabolism and were predominantly enriched in the OBM group (Fig. 3). Interestingly, the PFKM and GPI DE-PCGs associated with the galactose and starch and sucrose metabolism pathways were regulated by maximum number of different DE-miRNAs including 4 DE-miRNAs for PFKM and 3 DE-miRNAs for GPI respectively.

## Discussion

Given that obesity is a complex disorder, a full mechanistic insight requires a coordinated multi-omic approach. Also, deciphering the molecular networks that distinguish obesity with metabolic complications from obesity without metabolic consequences may lead to the identification of critical biomarkers that can be the basis for prognostication and personalized obesity management. In this work, we developed and used an integrative bioinformatics pipeline to identify enriched metabolic pathways by integrating data from transcriptional signatures, microRNAs and circulating metabolites, to obtain a more holistic picture of the differential enrichment of metabolic pathways between OBO and OBM. The identified miRNAs, transcripts, and metabolites are associated with abdominal adiposity, dyslipidemia, insulin resistance, glucose intolerance, and a proinflammatory/pro-thrombotic state.

We identified differentially expressed miRNAs known to influence pathways that regulate metabolism of amino acids (alanine, aspartate, asparagine, glutamate, glutamine, arginine, creatinine and proline), amino acid sugars and nucleotide sugars as well as carbohydrates (glyoxylate, galactose and sucrose). Impaired insulin signaling, a well-known abnormality in the metabolic syndrome, leads to lower uptake of glucose in skeletal muscles, and an upregulation of hepatic gluconeogenesis resulting in hyperglycemia. Metabolic biomarkers of gluconeogenesis and glycolysis have also been reported as biomarkers of the metabolic syndrome and other cardiometabolic diseases [37]. Several dysregulated microRNAs in OBM (Additional file 2: Tables S1.1 and S1.2) involved in glucose metabolism have been previously associated with the metabolic syndrome [38–40]. Intriguingly, Mononen et al. found a negative association between hsa-miR-93-3p and HbA1c levels [41]. Elevated insulin secretion reflects increased insulin resistance that is observed in the OBM group [42, 43]. The microRNA, miR-135, specific for the pancreatic islets and suppressing glucose-induced insulin secretion [44] was upregulated OBM. Wang and colleagues reported that miR-1303 was significantly upregulated in those with type 2 diabetes and was higher in those with complications [45]. On the other hand, the upregulated GNE gene in the OBM group, which is a target of miR-1303, encodes an enzyme that initiates and regulates the biosynthesis of N-acetylneuraminic acid (NeuAc), a precursor of sialic acids. Additionally, the NANS gene, that is higher in OBM, encodes an enzyme called N-Acetylneuraminate Synthase that functions in the biosynthetic pathways of sialic acid. Browning et al. have demonstrated that elevated sialic acid was positively associated with the metabolic syndrome, independent of BMI in women [46]. Other studies indicate that elevated levels of Neu5Ac during coronary artery disease progression as well as myocardial injury [47, 48]. Furthermore, the GALT gene, which is upregulated in OBM, encodes the enzyme galactose-1-phosphate uridylyltransferase, responsible for converting ingested galactose to glucose. Of note, disturbed galactose metabolism in the elderly and humans with diabetes accelerates cataract formation [49]. Other genes that were found to be higher in OBM encode glycolytic enzymes including Glucose-6-Phosphate Isomerase (GPI), Phosphoglycerate Mutase 1 (PGAM1), Hexosaminidase A, also known as HEXA as well as Phosphoglucomutase 2 (PGM2). Also, COLGALT1, which was down-regulated in OBM, is essential for extracellular matrix reorganization and post-translational collagen glycosylation in the endoplasmic reticulum [50–52]. Although, bi-allelic variants of COLGALT1 have been associated with brain small vessel disease, to date there are no studies that have investigated its role in development of diabetes or cardiovascular complications that result as an outcome of long-standing metabolic syndrome [53]. In addition to insulin resistance, amino acid catabolism is accelerated in diabetes reflected by muscle wasting along with increased uptake of alanine by the liver for gluconeogenesis and the breakdown of branched chain amino acids in muscle [54, 55]. An increasing body of evidence has linked visceral adiposity, insulin resistance, and diabetes to plasma amino acid levels [56]. Yamaguchi et al. have reported on the usefulness of measuring the plasma free amino acid profiles in evaluating risk for the metabolic syndrome, diabetes, dyslipidemia, and hypertension in a large cohort [57].

Some genes involved in amino acids metabolism that were significantly dysregulated in OBM versus OBO including ARG1 gene encoding arginase I that hydrolyzes arginine to ornithine and urea. Our findings reveal a significant difference in multiple amino acids and their derivatives between OBM and OBO, including L-Glutamine, L-asparginine, D-glutamate and L-Arginine. In this line, sarcosine, an elevated metabolite in the OBM group, is an amino acid that forms as an intermediate in the metabolism of choline in the kidney and liver and is also formed in the hydrolysis of creatine. Notably, sarcosine has not been reported previously in the context of obesity with metabolic complications. Whether sarcosine levels can be used as an early novel marker for developing metabolic syndrome in individuals with obesity but without metabolic complications cannot be determined in our small cohort and thus warrants further investigation. Plasma di-methylglycine, which is an amino acid derivative, has been shown to be independently related to acute myocardial infarction [58]. In addition to other amino acids, lysine degradation is favored to produce Acetyl-CoA during gluconeogenesis in the hepatic mitochondria [59, 60]. Several enzymes are upregulated in hepatic mitochondria of the OBM group to facilitate Acetyl-CoA production (ALDH2, HADH, DSLT, ECHS1, ACAT1, ALDH1B1). Interestingly, miR-636 was identified to promote atherosclerosis development via SP1 and lysine degradation [61]. Altered liver homeostasis has been demonstrated in the metabolic syndrome. We found a higher expression of the ALDH2 gene encoding for aldehyde dehydrogenase 2 which is central to alcohol metabolism. ALDH2 genetic polymorphisms have been also linked with an increased risk of type 2 diabetes in patients with coronary artery disease [62]. We also checked the expression of PCGs in different tissue types to validate our results. Since the samples were taken from the blood serum there exists a high chance that the differentially expressed genes will correspond to higher expression in blood tissue. We searched the PCGs in the GTEx portal (https://gtexportal.org/) and observed that these genes were highly expressed across multiple tissue types (Additional file 1: Figure S3).

The identified miRNAs, transcripts, and metabolites that are associated with the 8 enriched metabolic pathways between OBM and OBO groups as listed in Table 2. Lysine degradation happens through formation of saccharopine and the pipecolic acid pathway. The tissue-specific roles of these two pathways are still under investigation [63]. Morevoer, lysine degradation is favored to produce Acetyl-CoA during gluconeogenesis in the hepatic mitochondria [59, 60]. Amino sugar and nucleotide sugar metabolism, galactose metabolism, and starch and sucrose metabolism pathways mainly comprise of sugar metabolism and are associated with metabolic disorders. Arginine and proline metabolism pathway involves the biosynthesis and metabolism of several amino acids including arginine, ornithine, proline, citrulline, and glutamate. Alterations of arginine and proline pathway are associated with many disorders [64].

The strength of our study, in comparison to single omic studies, is that we integrated multi-omic data to deduce pathways differentially altered between OBO and OBM. Our data suggest early changes in key miRNAs and transcripts that have a widespread impact on the metabolism with functional consequences. The clinical significance of the identified network and cause or consequence of the involved elements remain to be validated in larger cohorts, while the biological roles of these elements in the metabolic syndrome can be further investigated for mechanistic insights. The intricate network revealed provides a panel of putative metabolic biomarkers with a comprehensive picture of the underlying biological processes as opposed to single omic studies. A limitation of our study is that the transcriptomic data from whole blood reflects blood cell function whereas the miRNAs and metabolites reflect functioning of all tissues. Despite this limitation, the integration of these data provided meaningful and metabolically relevant pathways that are differentially regulated between OBO and OBM; thus, metabolic pathways in circulating blood cells may reflect the metabolic status of most tissues. Future prospective studies can utilize these biomarkers to identify the predisposition and the sequence of occurrence of hypertension, dysglycemia and/or dyslipidemia in obesity which can accelerate the discovery of new personalized therapeutics.

## Supplementary Information

**Additional file 1: Figure S1.** Flowchart highlighting different number of MiRNAs, PCGs, Metabolites analyzed in our dataset and steps taken to identify key MiRNAs, PCGs, Metabolites involved in significantly enriched metabolic pathways between OBM vs OBO. **Figure S2.** Correlation between clinical traits and enriched metabolic pathways. Here each block represents correlation between the enrichment profile of a metabolic pathway and a clinical characteristic based on their values across the 39 participant profiles. Here we only considered those pathways which were significantly enriched and those clinical traits which were highlighted in Fig. 3A. **Figure S3.** Gene expression of 25 identified genes (differentially expressed in the samples) across multiple tissue types in the GTEx portal. (https://gtexportal.org/). The expression of these genes is higher across multiple tissue types and not biased towards blood tissue from which samples were collected.

**Additional file 2: Table S1**: List of significantly upregulated miRNAs in OBM compared to OBO. Here RQ refers to Relative Quantification measure using the standard formula [24]. An RQ value showcases the fold-change (FC) of a specific miRNA in two populations. An RQ=1 indicated that a specific miRNA was not differentially expressed in OBM versus OBO samples. Only those miRNAs were considered which were above the limit of quantification (LOQ). **Table S2.** List of significantly downregulated miRNAs in OBM compared to OBO. Here RQ refers to Relative Quantification measure using the standard formula [24]. An RQ value showcases the fold-change (FC) of a specific miRNA in two populations. An RQ=1 indicated that a specific miRNA was not differentially expressed in OBM versus OBO samples. Only those miRNAs were considered which were above the limit of quantification (LOQ). **Table S2.1. **List of metabolites significantly upregulated in OBM compared to OBO. The estimate represents the coefficients of logistic regression used to fit the model. The magnitude of a coefficient indicates the contribution of the metabolite in classifying OBM from OBO. The corresponding p-values of each metabolite are listed in the last column.** Table S2.2.** List of metabolites significantly downregulated in OBM compared to OBO. The estimate represents the coefficients of logistic regression used to fit the model. The magnitude of a coefficient indicates the contribution of the metabolite in classifying OBM from OBO. The corresponding p-values of each metabolite are listed in the last column.
