# Supplementary figures and images for: An integrated multi-omic approach demonstrates distinct molecular signatures between human obesity with and without metabolic complications: a case–control study
Source: J Transl Med. 2023 Mar 29;21:229. doi: 10.1186/s12967-023-04074-x (PMC10053148; doi:10.1186/s12967-023-04074-x)

## Slide 1
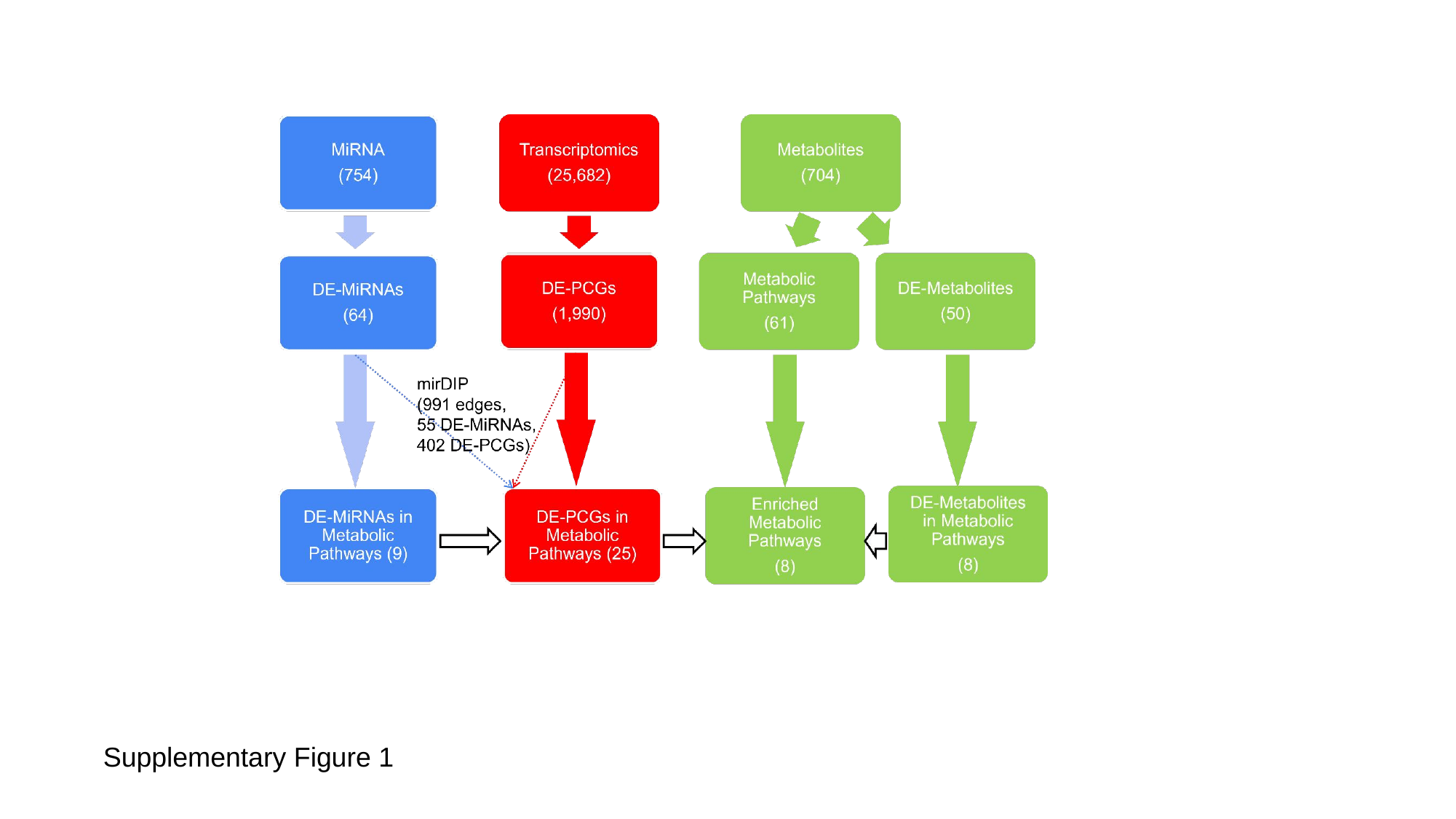

Supplementary Figure 1

## Slide 2
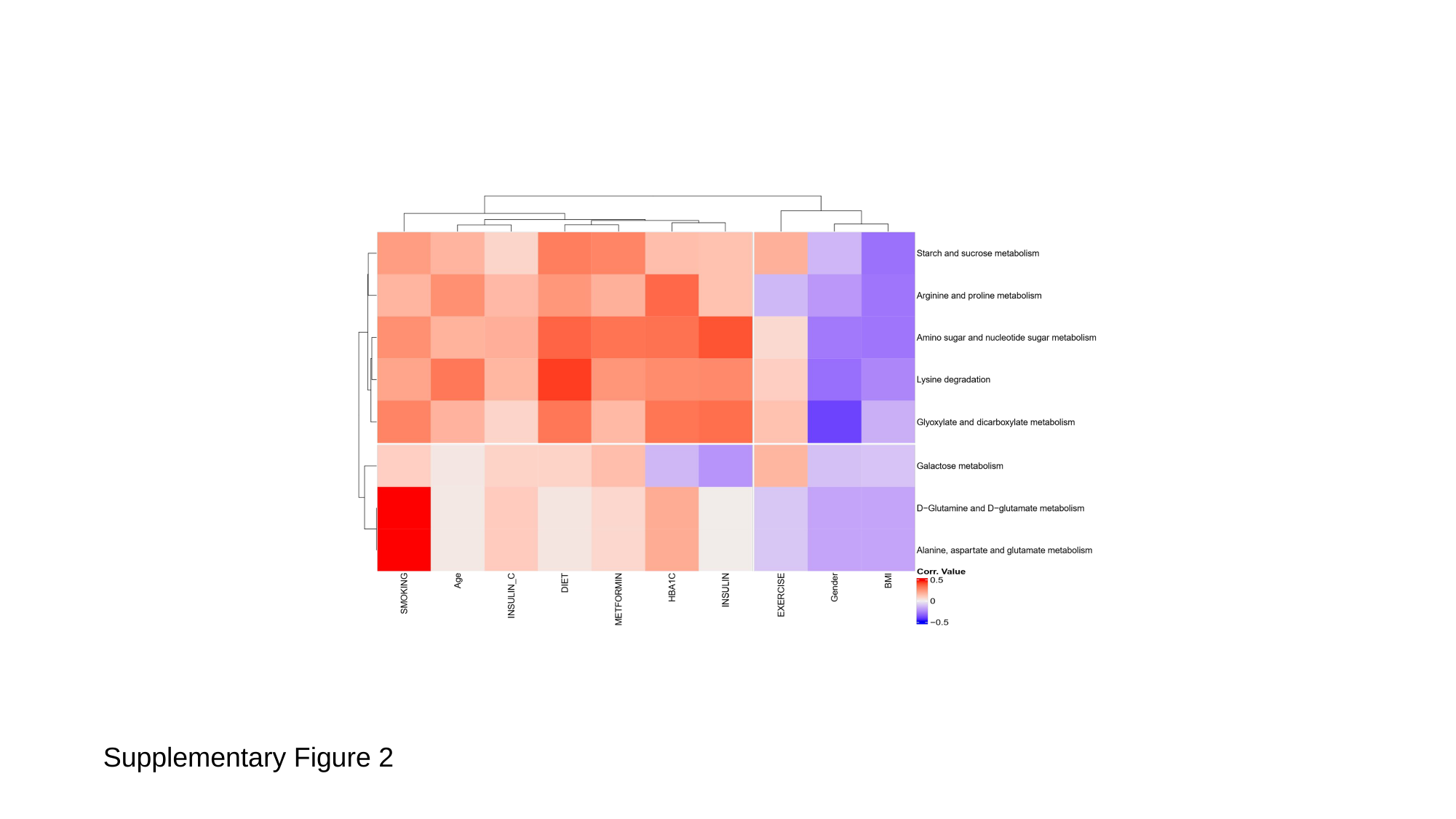

Supplementary Figure 2

## Slide 3
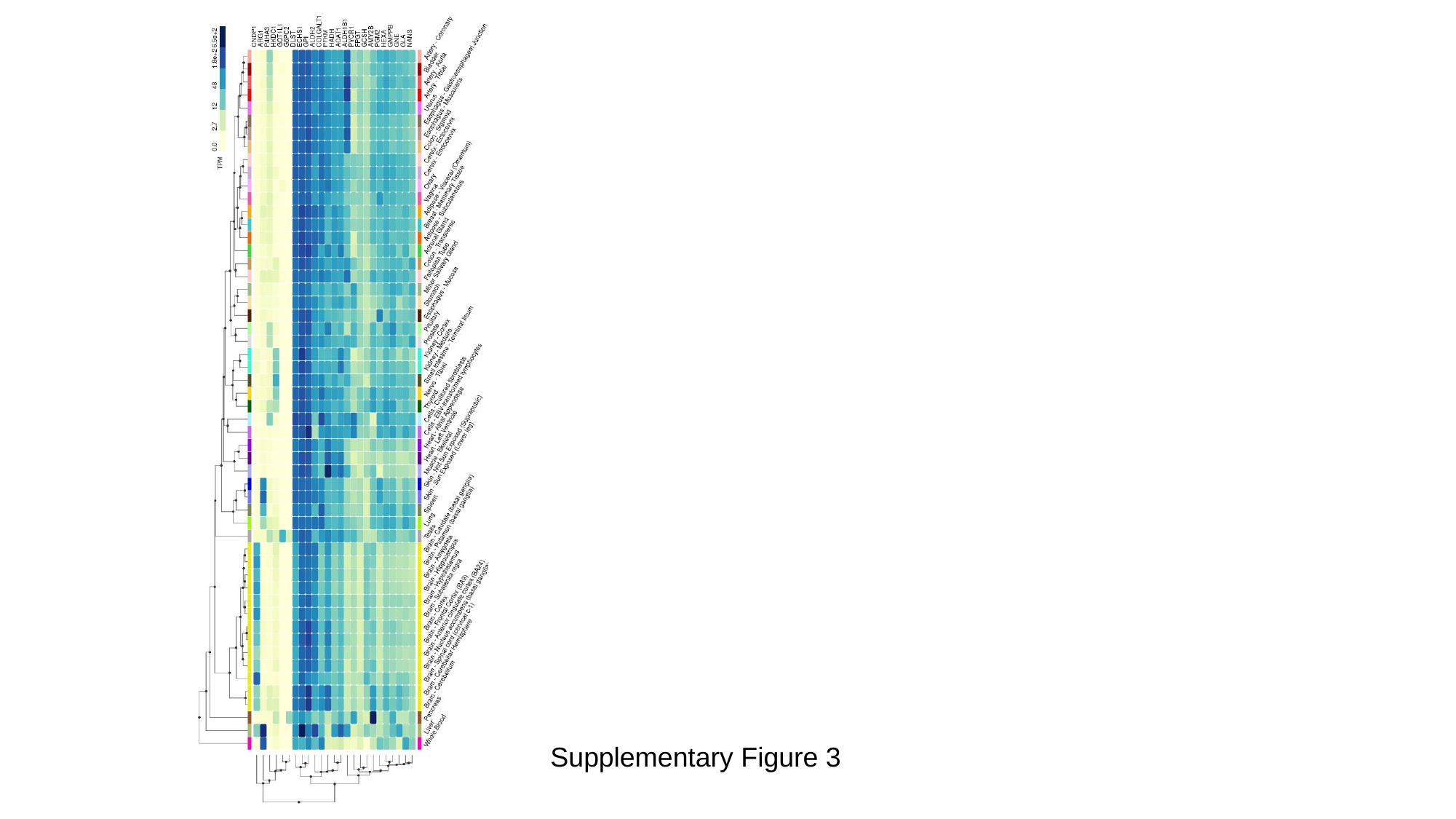

Supplementary Figure 3

Supplement: Supplementary file 1 — Additional file 1: Figure S1. Flowchart highlighting different number of MiRNAs, PCGs, Metabolites analyzed in our dataset and steps taken to identify key MiRNAs, PCGs, Metabolites involved in significantly enriched metabolic pathways between OBM vs OBO. Figure S2. Correlation between clinical traits and enriched metabolic pathways. Here each block represents correlation between the enrichment profile of a metabolic pathway and a clinical characteristic based on their values across the 39 participant profiles. Here we only considered those pathways which were significantly enriched and those clinical traits which were highlighted in Fig. 3A. Figure S3. Gene expression of 25 identified genes (differentially expressed in the samples) across multiple tissue types in the GTEx portal. (https://gtexportal.org/). The expression of these genes is higher across multiple tissue types and not biased towards blood tissue from which samples were collected. [file 12967_2023_4074_MOESM1_ESM.pptx]
